# Supplementary material for: Three-dimensional ℤ topological insulators without reflection symmetry
Source: Sci Rep. 2024 Feb 21;14:4288. doi: 10.1038/s41598-024-54821-3 (PMC10882006; doi:10.1038/s41598-024-54821-3)
Supplement: Supplementary file 1 — Supplementary Information. [file 41598_2024_54821_MOESM1_ESM.pdf]

Supplementary Material: Three-dimensional $\mathbb{Z}$ topological insulators without reflection symmetry

Alexander C. Tyner[1,2] and Vladimir Juričić[3,1]
[1] Nordita, KTH Royal Institute of Technology and Stockholm University, Hannes Alfvéns väg 12, 106 91 Stockholm, Sweden
[2] Department of Physics, University of Connecticut, Storrs, Connecticut 06269, USA and
[3] Departamento de Física, Universidad Técnica Federico Santa María, Casilla 110, Valparaíso, Chile
(Dated: November 30, 2023)

CLASS AIII, TWO-DIMENSIONAL $\mathbb{Z}$ TOPOLOGICAL BRANES

While the main body concentrates on three-dimensional insulators belonging to class AII due to their connection with physical spinful systems, it is informative to explore further classes within the Altland-Zirnbauer table of projected topological branes shown in the main body. Following traditional ten-fold classification, class AIII insulators support $\mathbb{Z}$ classification only in three-dimensions[1–6]. In this appendix, we exemplify the $\mathbb{Z}$ classification of a class AIII topological brane in two dimensions.

For clarity, we employ the same lattice tight-binding model utilized in the main body which is defined on a cubic lattice with the lattice parameter set to unity. The Bloch Hamiltonian takes the form, $H(\mathbf{k}) = \sum_{j=1}^{5} d_j(\mathbf{k})\Gamma_j$. Employing the basis,

$$\Gamma_{j=1,2,3} = \tau_1 \otimes \sigma_j,\ \Gamma_4 = \tau_2 \otimes \sigma_0,\ \Gamma_5 = \tau_3 \otimes \sigma_0, \tag{S1}$$

where $\tau_{0,1,2,3}(\sigma_{0,1,2,3})$ are the 2×2 identity matrix and three Pauli matrices, respectively, acting on the orbital (spin) degrees of freedom, the vector $\mathbf{d}(\mathbf{k})$ reads,

$$d_{j=1,2,3}(\mathbf{k}) = t_p \sin k_j,\ d_5(\mathbf{k}) = t_s(\Delta - \sum_{j=1}^{3} \cos k_j)\Gamma_5. \tag{S2}$$

In this model $t_{p,s}$ have units of energy and Δ is a dimensionless, non-thermal band parameters used for driving topological phase transitions. As class AIII systems support only chiral (unitary particle-hole) symmetry, we consider the Hamiltonian of the form,

$$H(\mathbf{k}) = \sum_{j=1}^{5} d_j(\mathbf{k})\Gamma_j + V(\mathbf{k}), \tag{S3}$$

where we define,

$$V(\mathbf{k}) = \alpha_1 \tau_1 \otimes \sigma_0 + \alpha_2 \tau_3 \otimes \sigma_3. \tag{S4}$$

In this model, the chiral symmetry, S, is generated by $S = \Gamma_4$ such that,

$$S^{-1}HS = -H. \tag{S5}$$

We fix $\alpha_{1,2} = 0.05$ such that these are weak perturbations and the bulk invariant is unaffected by their introduction. The bulk topology of this model is studied in Ref. [7], where it is shown that the bulk invariant is calculated as,

$$\nu(\Delta) = \frac{1}{48\pi^2} \int d^3k \epsilon^{ijl} \mathrm{Tr}[SH^{-1}(\partial_{k_i} H)H^{-1}(\partial_{k_j} H)H^{-1}(\partial_{k_l} H)], \tag{S6}$$

where ϵ^{ijl} is the Levi-Civita anti-symmetric tensor and S is the chiral symmetry operator. For the current model, this results in $\nu(\Delta = 2) = +1$ and $\nu(\Delta = 0.5) = -2$.

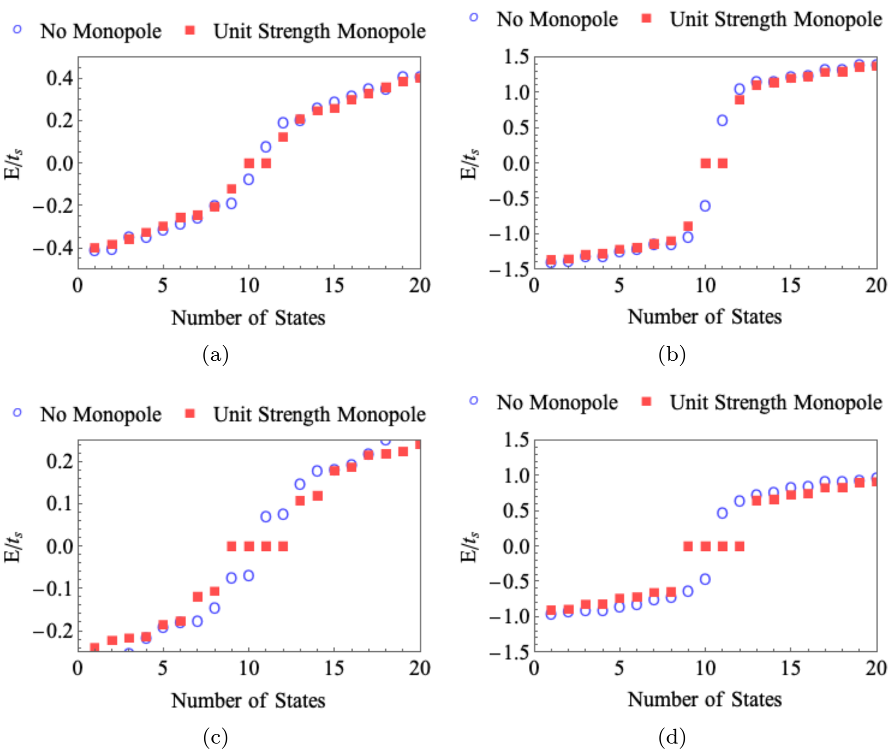

FIG. S1. Lowest lying twenty states for the three-dimensional, chiral-symmetric (class AIII) lattice model given in Eq. (S3) for (a) $\Delta = 2$ and (c) $\Delta = 0.5$. The spectra are plotted with and without the unit strength monopole inserted at the origin of the 10^3 lattice. We note that the monopole induces a number of zero modes given precisely by the relation, $\mathcal{N}_0 = 2|\nu(\Delta)|$. The number of zero modes remains intact when examining the spectra of the projected topological brane formed from the parent lattice for (b) $\Delta = 2$ and (d) $\Delta = 0.5$, confirming the $\mathbb{Z}$-classification of the two-dimensional brane.

A PTB is now constructed from the parent three-dimensional model following the procedure shown in the main text using a $10 \times 10 \times 10$ lattice. The lattice structure of the resulting two-dimensional brane is displayed in Fig. 2 of the main text. To probe the bulk topology, we utilize the corresponding real-space probe, as discussed in the main text, the magnetic monopole[8–14]. A magnetic monopole in inserted at the origin of three-dimensional lattice with the bulk invariant determined by the number of zero energy states upon monopole insertion.

Utilizing open boundary conditions in the three-dimensional parent model, we find the results shown in Fig. (1(a)) and Fig. (1(c)) for $\Delta = 2$ and $\Delta = 0.25$ respectively. These figures detail that the number of zero modes, $\mathcal{N}_0$, is given precisely by the relation, $\mathcal{N}_0 = 2|\nu(M)|$. In Fig. (1(b)) and Fig. (1(d)), the lowest lying modes of the PTB for $\Delta = 2$ and $\Delta = 0.25$ are shown, respectively. In each case the number of zero modes is unchanged with respect to the $d = 3$ dimensional parent model. This result illustrates the $\mathbb{Z}$ classification of PTBs belonging to class AIII in $d = 2$.

[1] C.-K. Chiu, J. C. Y. Teo, A. P. Schnyder, and S. Ryu, Rev. Mod. Phys. **88**, 035005 (2016).
[2] X.-L. Qi and S.-C. Zhang, Rev. Mod. Phys. **83**, 1057 (2011).
[3] S. Ryu, A. P. Schnyder, A. Furusaki, and A. W. Ludwig, New Journal of Physics **12**, 065010 (2010).
[4] A. P. Schnyder, S. Ryu, A. Furusaki, and A. W. W. Ludwig, Phys. Rev. B **78**, 195125 (2008).
[5] C.-K. Chiu, H. Yao, and S. Ryu, Phys. Rev. B **88**, 075142 (2013).
[6] T. Morimoto and A. Furusaki, Phys. Rev. B **88**, 125129 (2013).
[7] D. Liu, P. Matveeva, D. Gutman, and S. T. Carr, Phys. Rev. B **108**, 035418 (2023).
[8] E. Witten, Phys. Lett. B **86**, 283 (1979).

[9] H. Yamagishi, Phys. Rev. D **27**, 2383 (1983).
[10] H. Yamagishi, Phys. Rev. D **28**, 977 (1983).
[11] Y. M. Shnir, *Magnetic monopoles* (Springer Science & Business Media, 2006).
[12] G. Rosenberg and M. Franz, Phys. Rev. B **82**, 035105 (2010).
[13] Y.-Y. Zhao and S.-Q. Shen, arXiv:1208.3027 (2012).
[14] A. C. Tyner and P. Goswami, arXiv:2206.10636 (2022).
